
JOURNAL INFORMATION
==============================
NLM Title Abbreviation: Gastroenterol Rep (Oxf)
Journal ID: Gastroenterol Rep (Oxf)
NLM Journal ID: 101620508
Journal ID: gastro

Gastroenterology Report

EISSN: 2052-0034
Publisher: Oxford University Press

ARTICLE INFORMATION
==============================
PMCID: PMC6101495
DOI: 10.1093/gastro/goy032
Article ID: goy032
Article version: 1
Subjects: Chinese Abstracts

Chinese Abstracts – Volume 6 Issue 3*

Print publication date: 2018 Aug
Electronic publication date: 2018 Aug 10
Volume: 6
Issue: 3
First page: C1
Last page: C3
Copyright: © The Author(s) 2018. Published by Oxford University Press and Sixth Affiliated Hospital of Sun Yat-Sen University
Copyright year: 2018
License: This is an Open Access article distributed under the terms of the Creative Commons Attribution License (http://creativecommons.org/licenses/by/4.0/), which permits unrestricted reuse, distribution, and reproduction in any medium, provided the original work is properly cited.
License URL: https://creativecommons.org/licenses/by/4.0/

Page count: 3

==============================
*Gastroenterology Report*, 2018, 1–3

doi: 10.1093/gastro/goy032

ABSTRACTS

### Chinese Abstracts – Volume 6 Issue 3*

### Review（综述）

### Clostridium difficile colitis and zoonotic origins—a narrative review

### 艰难梭菌性结肠炎和动物源性：叙述性综述

[摘要] 艰难梭菌感染（CDI）是住院相关腹泻的主要原因, 严重时可导致假膜性结肠炎和中毒性巨结肠。在过去几十年中， CDI发生率显著升高, 2011年美国有45.3万例CDI病例。这与抗生素选择性压力、正常宿主肠道微生态的失衡以及抗生素抵抗艰难梭菌属的出现有关。随着在过去被认为很少发生CDI的人群中（如青年女性和从未接受抗生素治疗者），CDI被不断报道，社区获得性CDI的医疗负担不断增加。家畜、肉制品、海鲜及色拉中已被证实存在艰难梭菌。因此推测， 农产品中的艰难梭菌可能导致人类CDI的发生。艰难梭菌芽孢可在整个环境中传播, 家院、草坪、公共空间受到广泛污染, 从而为社区CDI提供了一个潜在的污染源。在澳大利亚, 主要的艰难梭菌感染种类是猪来源的UK PCR ribotype 014/020。本文主要讨论医院获得性CDI与社区获得性CDI的流行病学差异, 包括一些认为社区获得性CDI可能是一种人畜共患病的最新证据。

关键词：艰难梭菌；健康相关的感染；动物传染病

### Inflammatory caspase-related pyroptosis: mechanism, regulation and therapeutic potential for inflammatory bowel disease

### 炎症相关caspase介导的细胞焦亡：机制、调控及对炎症性肠病的潜在治疗价值

[摘要] 作为程序性细胞死亡的一种新的形式，细胞焦亡是由细胞内病原体感染诱发的炎症反应。如果细胞焦亡被过度激活， 会导致一些代谢性疾病、动脉粥样硬化以及重要器官损害。巨噬细胞是介导细胞焦亡的主要细胞， 其模式识别受体可以识别宿主细胞胞质溶胶内的危险信号和病原微生物。炎症相关caspase的激活可诱发细胞焦亡并产生促炎细胞因子，如IL-1β和IL-18。炎性程序性细胞死亡基于炎症相关caspase的不同可分为经典途径和非经典途径, 前者包括caspase-1（人类和小鼠）, 后者包括caspase-11（小鼠）或caspase-4和-5（人类）。炎症相关caspase的激活可切割成孔效应蛋白gasdermin-D，引起细胞渗透压的改变，继而导致胞膜破裂。炎症相关caspase可能成为炎症性肠病的潜在治疗靶点， 而细胞焦亡在其中具有重要作用。本文对细胞焦亡的机制进行综述， 并重点探讨了炎症相关caspase的调控及其对炎症性肠病的潜在治疗价值。

关键词：细胞焦亡；Gasdermin-D；炎性体；Caspase；炎症性肠病

### Original article（论著）

### Extra-pancreatic complications, especially hemodialysis predict mortality and length of stay, in ICU patients admitted with acute pancreatitis

### 胰外并发症尤其是血液透析可预测ICU急性胰腺炎患者的病死率和住院时间

[摘要] 背景和目的：入住ICU的急性胰腺炎患者因疾病严重和住院时间延长, 胰外并发症风险增高。我们旨在评估ICU急性胰腺炎患者胰外并发症发生率及其对住院时间和病死率的影响。

方法：本研究为回顾性队列研究, 研究对象来自一家三级转诊中心的287例ICU急性胰腺炎患者， 其中163例满足纳入标准入组研究。计算胰外并发症发生率， 并通过单因素和多因素分析评估胰外并发症对住院时间和病死率的影响。

结果：163例患者共出现158例次胰外并发症, 平均每例患者出现0.97种胰外并发症。95例患者出现至少1种胰外并发症, 另外68例未出现胰外并发症。在排除胰腺局部并发症患者后， 胰外并发症患者住院时间较无并胰外发症患者显著延长（14.7天 vs. 8.8天, $P < 0.01$）。非感染性胰外并发症患者病死率显著增高（24.0% vs. 16.2%, $P = 0.04$）。多因素分析结果显示, 需要透析是住院时间（IRR = 1.73, 95% CI:1.2632.378, $P < 0.01$）和病死率（IRR = 1.50, 95% CI:1.6236.843, $P < 0.01$）的独立预测因素。同时, 冠脉事件也是病死率的一个预测因素（$P = 0.05$）。而其他胰外并发症的预测价值未获证实。

结论：ICU急性胰腺炎患者常会发生胰外并发症， 从而影响住院时间。非感染性胰外并发症还会增加病死率。在消除了胰腺局部并发症的干扰后, 需要透析治疗被证实为ICU急性胰腺炎患者住院时间延长和病死率增高的独立预测因素。

关键词：重症急性胰腺炎；胰外并发症；重症监护室；住院时间；病死率；感染

*This article abstract was originally published in English. This translation has not been verified and should not be relied upon—it is provided for reference purposes only. The Publishers have not checked this translation and accept no liability for completeness or accuracy of this translation or the use of this translation for whatever purpose. This translation may be incomplete and inaccurate in whole or in part. If you need to rely upon a translation of this abstract, a professional human translator should be engaged to supply an accurate translation of the original English. When referencing articles from this journal, please always refer to the original English version, rather than a translated equivalent.

本文摘要最初是以英文发表。中文摘译未获认证，谨供参考。出版社没有对中文摘译进行核验，不负责译文的完整性和准确性，也不会将其用以其他目的。中文摘译可能会出现部分不完整或不准确的情况。如果你需要准确获悉摘要，最好能请一位专业译者针对英文原文进行翻译。阅读杂志文章，请参考英文原文，不推荐中文翻译。

### Gender, medication use and other factors associated with esophageal motility disorders in non-obstructive dysphagia

### 性别、药物应用及糜烂性食管癌可预测非梗阻性吞咽困难患者中食管动力障碍性疾病的罹患情况

[摘要] 背景：高分辨率食管测压（HREM）是非梗阻性吞咽困难的一种诊断方法。目前关于食管动力障碍预测因素的研究非常有限。因此，本研究旨在分析非梗阻性吞咽困难患者中食管动力障碍性疾病的患病率及其影响因素。

方法：对2014年1月1日至2015年12月31日期间在一家三级转诊中心接受HREM检查的所有非梗阻性吞咽困难患者进行回顾性分析。在获得伦理批准后（审批号：16-051），审阅临床记录，提取人口统计学资料、症状、药物应用史、上消化道内镜报告及食管pH检测结果。按照芝加哥标准对HREM测压结果进行判读。本研究主要结局指标是食管动力障碍性疾病的患病率，次要结局指标是其预测因素。

结果：155例非梗阻性吞咽困难患者纳入研究，平均年龄（55˘ 16）岁，其中72%为女性。HREM结果提示：49%的患者食管动力正常，无效食管动力者20%，无收缩者7.1%，II型贲门失弛缓者5.8%，流出道梗阻者5.2%，高收缩食管者4.5%，远端食管痉挛者3.9%，片段蠕动者1.9%，I型贲门失弛缓者1.9%，I型贲门失弛缓者0.6%。男性患者贲门失弛缓发生概率是女性的5倍（OR = 5.3, 95% CI：2.014.2；P = 0.001）；而糜烂性食管炎（OR = 2.9, 95% CI：1.17.7；P = 0.027）或使用钙通道拮抗剂者（OR = 3.0, 95% CI：1.27.4；P = 0.015），其低食管动力性疾病的发生概率增加3倍。

结论：按芝加哥标准，有49%的非梗阻性吞咽困难患者具有正常的食管动力。对于男性、糜烂性食管炎或使用钙通道拮抗剂的非梗阻性吞咽困难患者，应警惕食管动力障碍性疾病的可能。

关键词：食管吞咽困难；高分辨率食管测压；食管动力障碍性疾病

### Oxaliplatin-containing adjuvant chemotherapy improves the survival of locally advanced rectal cancer patients with pathological complete response after pre-operative chemoradiotherapy

### 包含奥沙利铂的辅助化疗可令术前放化疗后病理完全缓解的局部进展期直肠癌患者生存获益

[摘要] 背景：对于术前放化疗后获得病理完全缓解（pCR）的局部进展期直肠癌患者，术后辅助化疗的必要性仍不明确。本研究旨在探讨辅助化疗在这些患者中的治疗价值。

方法：回顾性分析2008年－2014年间一家综合癌症中心连续收治的105例经术前放化疗后获得pCR的局部进展期直肠癌患者的临床资料。围手术期化疗采用包含奥沙利铂和卡培他滨的XELOX方案。比较术后加行辅助化疗与未行辅助化疗两组患者的无瘤生存和总体生存

结果：83例（79.0%）例患者术后接受辅助化疗，22例（21.0%）未行辅助化疗。中位随访49月，辅助化疗组较未辅助化疗组3年无瘤生存率（92.8% vs. 86.4%, P = 0.029）和3年总生存率（95.1% vs. 86.1%, P = 0.026）显著提高。多因素分析显示，辅助化疗是无瘤生存的独立预后因素（HR = 0.271, 95% CI: 0.0800.916），但对总体生存并无显著影响。亚组分析显示，对于年龄≤60岁的患者，辅助化疗的生存改善作用更为显著（HR = 0.106, 95% CI: 0.0190.606；P = 0.012）。

结论：包含奥沙利铂的辅助化疗可以为pCR直肠癌患者带来生存获益，尤其是对于年轻患者。但其是否应常规用于pCR患者尚需进一步研究证实。

关键词：辅助化疗；直肠癌；术前放化疗；病理完全缓解；生存

### The added clinical value of performing CT colonography in patients with obstructing colorectal carcinoma

### CT结肠成像应用于梗阻性结直肠癌的临床价值

[摘要] 背景：光学肠镜检查时会有一小部分因肿瘤梗阻无法完成。按目前指南，CT结肠成像（CTC）可用于防止遗漏同时性肿瘤。本研究旨在评估CTC发现同时性肿瘤的概率以及改变外科治疗策略的比例。

方法：在这项回顾性研究中，267例患者因梗阻性结直肠癌未完成光学肠镜的检查而行CTC检查。其中，210例接受外科手术的患者纳入研究。回顾性分析入组患者的光学肠镜报告、CTC报告及手术记录，以术中所见和术后肠镜作为金标准，评估同时性肿瘤的出现概率。

结果：210例患者中有6例（2.9%）经CTC检查提示梗阻近端存在同时性肿瘤。其中3例同时性结直肠癌经手术证实，这3例同时性结直肠癌的发现均导致了手术方式的改变。另外3例同时性肿瘤均为大的良性息肉，但由于这些息肉位于梗阻肿瘤同一肠段，故未改变原始手术方式。

结论：在梗阻性结直肠癌中，梗阻近端同时性结直肠癌的出现概率较低。有1.4%的梗阻性结直肠癌患者会因CTC检查发现同时性肿瘤而导致手术方式的改变。有必要将CTC作为梗阻性结直肠癌的常规检查手段。

关键词：CT结肠成像；光学肠镜；结直肠癌；同时性肿瘤；术前评估

### Unexplained iron deficiency anemia: does Helicobacter pylori have a role to play?

### 不明原因的缺铁性贫血：是幽门螺杆菌导致的吗？

[摘要] 背景和目的：尽管缺乏证据支持，但幽门螺杆菌（*Hp*）的检测还是被推荐用于不明原因缺铁性贫血（IDA）的诊断。本研究旨在研究无明显上消化道出血表现的成年人群中，*Hp*感染与不明原因的铁缺乏症（ID）或IDA之间的关系。

方法：本研究为回顾性单中心队列研究。1253例行食管胃十二指肠镜检查的连续病例中，523例未见明显的消化道出血表现或IDA危险因素的患者纳入研究。采用Fisher精确概率检验、卡方检验和独立样本*t*检验对*Hp*阳性与*Hp*阴性患者进行比较。采用单因素和多因素逻辑回归分析来明确ID/IDA的危险因素。

结果：523例病例中中103例（19.7%）*Hp*阳性，420例（80.3%）*Hp*阴性。307例患者进行了血清铁检测，其中68例（22.1%）诊断为不明原因的ID；510例患者进行了血红蛋白检测，其中28例（5.4%）诊断为不明原因的IDA。单因素和多因素逻辑回归分析结果显示，*Hp*感染与ID/IDA之间并无显著相关性。

结论：在无消化性溃疡病或明显上消化道出血的成年人群中，我们并没有发现*Hp*感染与ID/IDA之间的相关性。

关键词：幽门螺杆菌；贫血；铁缺乏症

### Vedolizumab in the treatment of Crohn's disease of the pouch

### Vedolizumab治疗储袋克罗恩病

[摘要] 背景：我们最近研究显示，vedolizumab治疗慢性抗生素难治性储袋炎安全有效。然而，尚未见其应用于储袋克罗恩病（CD）的报道。本研究旨在评估vedolizumab治疗储袋CD的疗效和安全性。

方法：本组储袋CD病例来自我们的前瞻性储袋炎数据库中2015－2017年间的病例。采用改良储袋炎疾病活动指数（mPDAI）评估储袋患者疾病活动度，mPDAI包括3个变量：症状（06分），内镜所见（06分），组织学评估（06分），总分18分。治疗前和治疗后（至少6个月），通过储袋镜检查和门诊随访来进行mPDAI评分。

结果：12例因药物难治性溃疡性结肠炎（UC）而行结直肠切除、回肠储袋肛管吻合的患者纳入研究。肠切除术前UC诊断平均年龄（25.0˘ 11.5）岁。目前平均年龄（41.0˘ 12.1）岁，其中女性9例（75.0%），吸烟者3例（25.0%），既往接受抗TNF药物治疗者8例（66.7%）。平均vedolizumab治疗时间（1.0˘ 6.4）年，治疗后mPDAI症状评分显著降低（3.50 1.93 vs. 5.08˘ 0.79, P = 0.015）。治疗前后mPDAI内镜评分：输入袢（1.25˘ 1.36 vs. 0.91˘ 1.50, P = 0.583）；储袋体（2.58˘ 1.68 vs. 2.27˘ 2.05, P = 0.701）；直肠套袖（2.67˘ 1.93 vs. 2.02˘ 2.12, P = 0.511）。vedolizumab治疗期间无一例患者出现不良反应。

结论：本研究显示，vedolizumab治疗储袋CD似乎是安全有效的，可减轻患者症状。

关键词：抗肿瘤坏死因子制剂；克罗恩病；结直肠切除术；vedolizumab

### Three-cavity clearance (TCC) can decrease the fistula rate after drainage of a perianal abscess: a case–control study

三腔间隙引流（TCC）可降低肛周脓肿术后肛瘘发生率：一项匹配病例对照研究

[摘要] 目的：本研究目的旨在评估三腔间隙引流（TCC）治疗肛周脓肿的安全性和有效性。

方法：收集2013年6月至2016年3月间在南京中医药大学附属第二和第三医院的肛周脓肿患者的病例资料，其中，46例患者接受了TCC治疗。按照性别、年龄、脓肿部位进行1:1:1匹配，选取同期行单纯切开引流术的46例患者和行一期切开引流挂线术（根治性手术）的46例患者进行对比分析。比较三组患者的住院时间、切口愈合时间、肛瘘发生率及肿门失禁情况。

结果：TCC组患者术后肛瘘发生率为13.0%，显著低于单纯切开引流组（39.1%, $P < 0.01$），与根治性脓肿切开组相当（8.7%, $P > 0.05$）。根治性脓肿切开组有2例患者术后出现肛门失禁、腹胀和污粪，其Wexner失禁评分分别为6分和3分；而TCC组和单纯切开引流组无肛门失禁发生。三组患者切口愈合时间和住院时间的差异均无统计学意义（均$P > 0.05$）

结论：TCC是一项安全有效的保留括约肌功能的肛周脓肿治疗术式，可降低脓肿引流术后肛瘘发生率。该术式有望在临床实践中推广应用，其临床疗效尚需进一步研究证实。

关键词：三腔间隙引流（TCC）；肛周脓肿；肛瘘；引流术；挂线术

### Case report（病例报告）

### Spontaneous rupture of intrahepatic pseudocyst into the inferior vena cava

肝内假性囊肿自发破裂溃入下腔静脉

### Cetirizine-induced hepatotoxicity: case series and review of the literature

西替利嗪引起的肝中毒：病例系列报道和文献复习

### A life-threatening duodenal ulcer hemorrhage due to previously unknown primary hyperparathyroidism

因既往未知的原发性甲状旁腺功能亢进引起的危及生命的十二指肠溃疡出血

### Technical note（技术交流）

### A novel anti-reflux reconstruction after laparoscopic total gastrectomy: jejunal pouch-esophageal anti-reflux anastomosis

腹腔镜全胃切除术后一项新的抗反流消化道重建方法：空肠储袋-食管抗反流吻合术

### Commentary（专家笔谈）

### A commentary on soft tissue segmentation methods during image-guided precise liver surgery

影像学引导的精准肝切除术中软组织分割方法

### Corrigendum（更正启示）
